# The Effects of Mesenchymal Stem Cell on Colorectal Cancer

**DOI:** 10.1155/2021/9136583

**Published:** 2021-07-24

**Authors:** Jintao Yuan, Zhiping Wei, Xinwei Xu, Dickson Kofi Wiredu Ocansey, Xiu Cai, Fei Mao

**Affiliations:** ^1^The People's Hospital of Danyang, Affiliated Danyang Hospital of Nantong University, Zhenjiang, Jiangsu 212300, China; ^2^Key Laboratory of Medical Science and Laboratory Medicine of Jiangsu Province, School of Medicine, Jiangsu University, Zhenjiang, 212013 Jiangsu, China; ^3^Directorate of University Health Services, University of Cape Coast, Cape Coast, Ghana

## Abstract

Colorectal cancer (CRC) is a common malignant tumor of the gastrointestinal tract with nonobvious early symptoms and late symptoms of anemia, weight loss, and other systemic symptoms. Its morbidity and fatality rate are next only to gastric cancer, esophageal cancer, and primary liver cancer among digestive malignancies. In addition to the conventional surgical intervention, other therapies such as radiotherapy and chemotherapy and new treatment methods such as biologics and microbiological products have been introduced. As a promising cell therapy, mesenchymal stem cell (MSC) has attracted extensive research attention. MSCs are early undifferentiated pluripotent stem cells, which have the common features of stem cells, including self-replication, self-division, self-renewal, and multidirectional differentiation. MSCs come from a wide range of sources and can be extracted from a variety of tissues such as the bone marrow, umbilical cord, and fat. Current studies have shown that MSCs have a variety of biological functions such as immune regulation, tissue damage repair, and therapeutic effects on tumors such as CRC. This review outlines the overview of MSCs and CRC and summarizes the role of MSC application in CRC.

## 1. Introduction

Since the discovery of MSCs, researchers have deeply discussed their physiological characteristics. According to the definition of the International Society for Cell & Gene Therapy (ISCT®) Mesenchymal Stromal Cell (ISCT MSC) committee, MSCs are different from mesenchymal stromal cells [1]. MSCs are heterogeneous cell groups with the ability of self-renewal and differentiation. Besides bone marrow-derived MSCs (BM-MSCs) and umbilical cord-derived MSCs (UC-MSCs), which are widely studied, there are several other sources of MSCs including peripheral blood, adipose tissue, and minced pulp tissues [2–5]. According to their donors and passages, MSCs derived from diverse sources have different surface markers. The ISCT committee suggests that the minimum criteria for identification is plastic adhesion, the ability to express CD73, CD90, and CD105, but not expressing CD11b, CD14, CD19, CD34, CD45, CD79a, and HLA-DR [1, 6].

Compelling evidence indicates that MSCs have great potential in regenerative medicine. MSCs can participate in tissue repair and regeneration [7], immune homeostasis regulation [8], inflammation resistance, and tumor inhibition [9, 10]. Exosomes derived from MSCs, as one of the paracrine products, have attracted researchers' attention recently because of their similar functions to MSCs. Exosomes, the 30-150 nm vesicles, are formed by endocytosis of cells, which encapsulate nucleic acids, proteins, lipids, and metabolites from the original cells when discharged from them [11, 12]. It has been found that exosomes can participate in information transmission between cells through packaged biologically active substances such as nucleic acid and protein [13, 14]. Through receptors and ligands, exosomes target specific cells and achieve information exchange by their integration into cell membranes [15]. Exosomes derived from MSCs possess the functional ability to be applied for tissue damage repair and cancer inhibition and avoid the adverse reactions associated with cellular therapy [16–18]. As a cell-free therapy, exosomes have great prospects in cancer treatment.

CRC is among the top five cancers in the world in morbidity and mortality. Although the specific pathogenic factors that cause CRC remain unclear, its incidence is related to heredity, smoking, and eating pickled products, among other environmental factors [19, 20]. CRC begins insidiously and is usually screened for with stool tests, radiology, blood tests, and colonoscopy [21, 22]. Traditional chemotherapy has a poor curative effect on CRC with associated high toxicity and drug resistance [23]. Researchers have found that natural compounds, such as resveratrol and curcumin can treat CRC without huge adverse reactions through a series of animal models [24]. However, the lack of clinical trials hinders the application of these compounds. MSCs and their derived exosomes possess the ability to regulate the tumor microenvironment through paracrine and direct contact to inhibit CRC cells [25, 26]. MSCs can also be used as adjuvant therapy for tumor treatment, where they enhance the tumor cell-cytotoxic effect of antitumor drugs via cytokine secretion [27]. In this review, we aim to discuss the current knowledge of the effect of MSCs and their exosomes on CRC, including the mechanism of MSCs' inhibitory function in the CRC microenvironment, and the tumor-promoting effect of MSCs that cannot be ignored. This will bring up-to-date data on the status of the effects of MSCs and MSC-derived exosomes on CRC.

## 2. Overview of MSCs

### 2.1. Sources of MSCs

MSC is favored by the field of regenerative medicine because of its ability of self-renewal and directional differentiation under specific regulations. The characteristics of MSCs differ according to their different sources. Studying the characteristics of MSCs may help better selection of the suitable source, regulate their differentiation, and perhaps reduce the side effects of clinical application.

The sources of MSCs are varied, among which BM-MSCs, UC-MSCs, and MSCs from adipose tissue (AD-MSCs) are the most widely explored and applied [2]. The bone marrow provides an abundant source of MSCs, although this sampling method is invasive and causes great pain to patients. Alternatively, femoral shaft MSCs obtained during total hip arthroplasty have similar characteristics to iliac crest aspirate MSCs and can secrete cytokines such as epidermal growth factor (EGF), fibroblast growth factor (FGF), and insulin-like growth factor (IGF) [28]. The method of extracting AD-MSCs is also invasive, while the success rate and colony frequency of isolating AD-MSCs are relatively high [2]. MSCs can also be separated from neonatal-related tissues, such as the umbilical cord, umbilical cord blood, placenta, and amniotic membrane [29]. MSCs derived from umbilical cord blood (UCB-MSCs) and UC-MSCs are separated from the umbilical cord-related tissue and are widely used because their collection causes no pain to patients. At the same time, UCB-MSCs have a relatively longer survival time and higher differentiation potential [2, 29]. MSCs from neonatal tissues have better application potential than those from adult tissues [30].

Other noninvasive sources of MSCs are MSCs derived from endothelium (eMSCs), Wharton's Jelly (WJ-MSCs), and menstrual blood (MenSCs), which are considered to regulate the innate and adaptive immune system both in vivo and in vitro [31, 32]. MSCs derived from pluripotent stem cells (PSCs) have been shown to overlap with BM-MSCs in gene expression and have similar immunomodulatory and inflammatory suppressive functions. Most importantly, they have the highest proliferation efficiency and longer passage time, which can be widely used as the source of MSCs [33]. MSCs derived from dental tissue, including dental pulp (dPSCs) [34], periodontal ligament (PDLSCs), gingiva (GSCs), apical papilla (APSCs) [35], and dental follicles (DFSCs)[10], have not been widely studied, while they have great potential in dental tissue regeneration. Sufficient research on the source of MSCs can help in the selection of suitable MSCs for specific disease treatment, establish a matching culture and separation system, and accelerate the development of MSC therapy. The sources and characteristics of MSCs are summarized in Table 1.

### 2.2. Differentiation of MSCs

One of the reasons MSCs are popular in the field of regenerative medicine is their multiple differentiation ability. MSCs have the ability to differentiate into osteoblasts, chondroblasts, and adipocytes and even develop a relatively mature induction system for different target cells [54, 55]. The regulation of MSCs differentiation is a complex system involving series of factors including cytokines, transcription factors, and nucleic acids. In theory, MSCs can be differentiated into cells through precise regulation [56]. For example, the osteogenic differentiation of MSCs could be active through calcium signaling [57]; miR-27a is involved in the differentiation of MSCs from osteogenesis to adipogenesis in postmenopausal osteoporosis [58]; transcription factors, including Runt-related transcription factor 2 (Runx2), SRY-related high-mobility group-box gene 9 (Sox9), the adipogenic-specific peroxisome proliferation-activated receptor *γ* (PPAR*γ*), the member of the helix-loop-helix family transcription factors, MyoD, and the GATA zinc finger transcription factor family, GATA4 and GATA6, play a significant role in the differentiation of MSCs as corroborated by Almalki and Agrawal [59]; intercellular adhesion molecule-1 (ICAM-1) has been shown to inhibit MSC differentiation into adipocytes by activating the extracellular signal-regulated kinase (ERK) pathway and maintain MSCs undergoing adipogenesis through the p38 pathway [60].

MSCs are recruited in injury repair, and their ability of multidirectional differentiation is used in the reconstruction of injured tissues, with most of such reports described in MSCs from autologous sources [61]. Allogeneic MSCs disappear soon after injection in vivo, which may limit their curative effect [62]. MSCs play a therapeutic role by secreting active factors, and their differentiation ability in vivo is rarely reported, although in vivo studies of MSCs in the tumor microenvironment have been explored [63]. In the tumor microenvironment, the differentiation of MSCs could result in tumor promotion. For example, the infiltration of multipotential MSCs was found during the transformation of human prostate tumor, which suggests a possible role of the multipotential differentiation in cancer promotion [64]. Researchers found that limiting MSCs' differentiation potential may become a new target for cancer treatment [65]. It is revealed that CXCR4/TGF-*β*1 can mediate the self-differentiation of human BM-MSCs into cancer-related fibroblasts (CAFs) in CRC, thus playing a role in promoting cancer [66]. Research focuses on the differentiation function of MSCs in CRC which is conducive to finding more cancer targets; hence, more related research is needed to explore the field in search good CRC treatment.

### 2.3. General Effect of MSCs

From inflammation, infection, abnormal metabolism, immune disorder, and tissue damage to the tumor, MSCs have obvious curative effects in the treatment of these diseases. It is generally believed that MSCs exert therapeutic effects through direct contact and paracrine action, where MSC-derived extracellular vehicles (EVs) are reported as the mechanism behind these effects [67, 68]. In the immunoregulatory process, monocytes can rapidly phagocytize MSCs injected in vitro, changing their phenotype, expressing interleukin- (IL-) 10 and programmed death ligand-1 (PDL-1), and reducing the expression of tumor necrosis factor- (TNF-) *α*. These monocytes can migrate through the whole body via the circulatory system and play a follow-up immunoregulation effect [69]. Macrophage phenotype could also respond to the effect of MSCs by switching from proinflammatory “M1” to anti-inflammatory “M2” [70]. TGF-*β* and polyethylene glycol- (PEG-) 2 secreted by MSCs complete this process through the Akt/FoxO1 pathway [71, 72]. At the same time, the direct contact between proinflammatory macrophages and MSCs can enhance the secretion of tumor necrosis factor-stimulated gene-6 (TSG-6), and the production of anti-inflammatory T cells and macrophages [73]. MSCs play a role in relieving pain, inhibiting inflammation, sustaining proliferation, and regenerating matrix and cartilage in the process of bone regeneration with the regulation of V-Akt murine thymoma viral oncogene homolog (AKT), ERK, and the serine/threonine kinase AMP-activated protein kinase (AMPK) signaling [74].

For the treatment of tumors, the role of MSCs is complex. Compelling evidence indicated that MSCs can play a role in the process of tumor suppression, tumor growth, and drug resistance. The microRNA- (miR-) 100 secreted by MSCs is related to the downregulation of vascular endothelial growth factor (VEGF) in tumor angiogenesis [75], and miR-23b promotes the dormancy of metastatic breast cancer cells [76]. However, microRNA does not always play a positive role in inhibiting tumors. This is confirmed by several studies including that of Wei et al., who reported that microRNA-375 increases the invasion and metastasis of CRC via regulating the target gene RECK [77]. The fusion of MSCs with benign tissue cells may be beneficial to the repair of tissue damage, but after MSCs are recruited into tumor tissues, the fusion with tumor cells may be related to tumor metastasis [78]. This means that the direct contact and paracrine pathway of MSCs may not be able to alleviate the tumor. Further studies are needed to explore the mechanism of MSCs' functional effects to enhance the antitumor application of MSCs and avoid the tumor-promoting effect.

### 2.4. General Clinical Application of MSCs

With the deepening of the understanding of the mechanism of MSCs' functions, their clinical application is gradually standardized, and their safety has been greatly improved. Therefore, clinical trials of MSCs are constantly carried out. Intravenous injection and intralesional injection account for the majority of MSCs administration. A three-year follow-up after intravenous infusion of UC-MSCs showed there were no abnormalities in blood routine, liver and kidney function, and immunoglobulin in the treatment of rheumatic immune diseases. Moreover, the health index and joint function index significantly improved [79]. For patients with multiple sclerosis, MSCs can improve the quality of life of patients without serious adverse reactions, and intrathecal administration is more effective than intravenous injection [80, 81]. More MSC-related clinical trials presented so far in the field of the cardiovascular system, digestive system diseases, nervous system, and endocrine system prove its effectiveness (Table 2).

The reduction of side effects in the clinical use of MSCs does not mean that it is completely safe. More attention should be paid to the safety research of MSCs application. Matthay et al., demonstrated that one dose of intravenous allogeneic BM-MSCs is safe in patients with moderate to severe acute respiratory distress syndrome (ARDS) [82]. The safety and efficacy of the intravenous infusion of UC-MSCs in patients with heart failure are also demonstrated by Bartolucci et al. [83]. Researchers using modified MSCs to treat gastrointestinal cancer found that MSCs were safe and well tolerated in patients with gastrointestinal cancer. However, due to the small number of patients and the heterogeneity of tumors, the treatment received by patients may affect the interaction between tumor microenvironment and MSCs; no improvement in tumor activity was observed [84]. Another group of researchers found no dose-limiting toxicity in MSC treatment of prostate cancer, while the effectiveness is still worth further exploring [85]. There are few clinical applications of MSCs in tumor treatment, which may be due to the lack of standard MSCs separation and infusion standards, the inability to inhibit the tumor-promoting effect of MSCs, the lack of cognition of MSCs homing mechanism, and the heterogeneity of the tumor. In the application of tumor therapy, there is a need to explore MSCs and tumors for a long time.

## 3. Overview of CRC

### 3.1. The Pathogenesis of CRC

CRC is a heterogeneous malignant tumor of the colon and rectum, the fourth most common cancer around the world contributing to 9.7% of the global cancer burden [108] and the third most frequent malignant tumor in China [109]. Bad living habits and environmental pollution contribute to the prevalence of CRC. It is reported that up to 90% of the morbidity risk of CRC is related to environmental factors such as diet [110]. CRC occurrence is linked with inappropriate dietetic habits such as high consumption of heme-iron foods and alcohol and low consumption of fruits, vegetables, fiber, fish, dairy products, and vitamin C. Furthermore, unhealthy lifestyles such as obesity and lack of exercise increase CRC risk [111].

**Figure 1:**
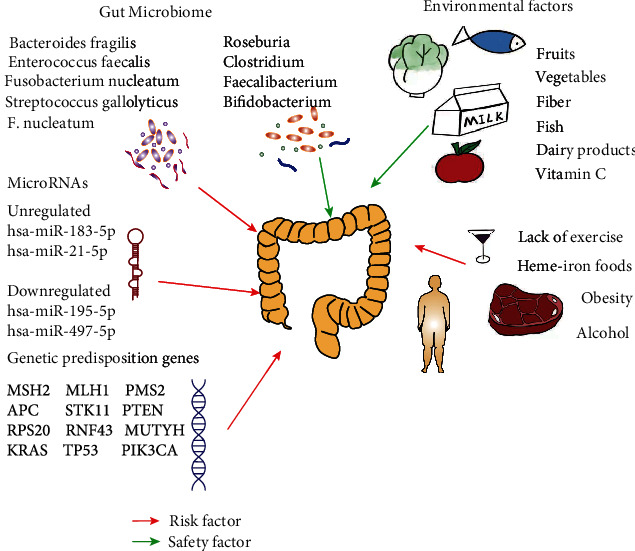
The influencing factors of CRC occurrence.

Besides diet and lifestyle, genetics and certain diseases can also contribute to CRC. In all CRCs, the genetic predisposition genes with high cancer risk are 2–8%. When the pathogenic mutations in high- and moderate-penetrance genes are added, the ratio will rise to 6–10% [112]. The risk of developing CRC increases in patients with inflammatory bowel disease (IBD), where the inflammation involved with IBD serves as a hazard for CRC onset [110, 113, 114]. Diabetes is positively relevant to the risk of CRC in men (HR = 1.17; 95% CI: 1.08-1.26; *I*^2^ = 0%) and women (HR = 1.13; 95% CI: 0.82-1.56; *I*^2^ = 46%) [115]. Microsatellite instability and microRNAs play important roles in the occurrence of CRC [114, 116]. Furthermore, intestinal microbiota highly impacts the state of CRC, where a proper balance in the diversity and composition positively correlates with a good CRC prognosis [117, 118]. The influencing factors of CRC occurrence are shown in Figure 1.

Individual habits such as eating red meat and consuming alcohol increase CRC risk, while eating dairy products, fish, and vitamin C-rich foods reduces the risk. Intestinal flora also plays a dual role in the development of CRC. Gene mutation and some noncoding RNAs can promote the occurrence of CRC.

### 3.2. Current Therapeutic Interventions in CRC

In addition to surgery, there are several current and emerging treatment options for CRC including chemotherapy, radiotherapy, targeted therapy, and immunotherapy, among others [119]. These CRC treatment interventions are summarized in Table 3.

## 4. MSC Studies on Tumors

**Figure 2:**
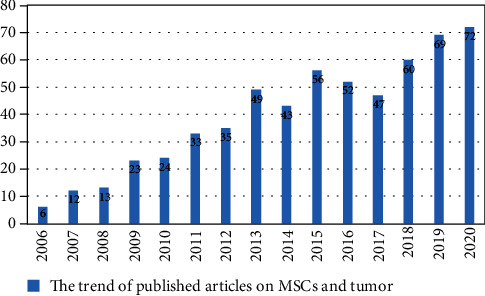
The trend of research on the application of MSCs in tumors.

**Figure 3:**
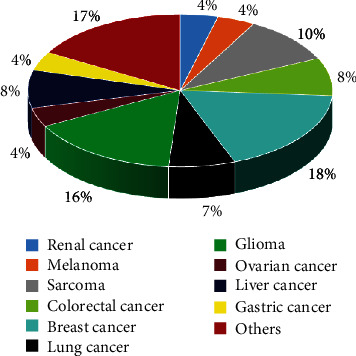
MSCs studies on the different kinds of tumors.

The biological functions of MSCs have been studied and applied in clinical trials, including the study of their effects on tumors. A search of the terms “mesenchymal stem cell and tumor” in the PubMed database revealed a total of 594 documents published in nearly 15 years, from January 1st, 2006, to December 31st, 2020 (Figure 2). Further statistical analysis of the MSC studies on different tumors in the last 15 years showed approximately 8% of literature reports on the role of MSCs on CRC (Figure 3).

The increasing attention given to MSC studies correlates with the rising application in cancer studies. Over the past few years, the number of published researches on the effect of MSCs on tumors has steadily been increasing.

This figure represents studies on MSCs and different tumors in the past 15 years, where CRC represents 8% of total studies. Those with a proportion less than 4% are classified as others.

## 5. The Effect of MSCs on CRC

According to the literature, MSCs possess a double-edged sword property on CRC. In effect, MSCs can significantly inhibit the proliferation, migration, and infiltration of tumor cells to prevent the occurrence and progression of CRC, while in other conditions, MSCs serve as promoting agents for CRC progression (Table 4).

### 5.1. Inhibitory Effects on CRC

Studies show that under certain treatment conditions, MSCs can inhibit the proliferation of CRC cells and promote apoptosis, thus inhibiting the progression of CRC. A study by Chen and colleagues indicates the hypothesis that MSCs improve tumorigenesis in IBD by inhibiting the expression of proinflammatory cytokines and activation of STAT3 [133]. In a study of azomethane- (AOM-) induced carcinogenesis, exogenous MSCs were demonstrated to possess inherent antitumor properties. Specifically, the MSCs could induce apoptosis by blocking the cell cycle in the G1 phase, and the intervention of MSCs could lead to the dysregulation of the Wnt and TGF-*β*-Smad signaling pathways in the body, thus interfering with tumor initiation [134]. In the study of Feng and colleagues, low doses of ultraviolet radiation and X-ray irradiation caused BM-MSCs to secrete specific cytokines (TNF-*α*, IFN-*γ*) to inhibit CRC cell proliferation and induce apoptosis, showing antitumor effects [26].

Moreover, MSCs can play a tumor-suppressive role through microRNAs contained in exosomes, a paracrine mode. Yan et al. presented that miR-16-5p overexpression in BM-MSC-derived exosomes inhibits the proliferation, migration, and invasion of CRC cells and promotes the apoptosis of CRC cells by downregulating ITGA2 expression [135]. Chen and colleagues also demonstrated in in vitro cell experiments that BMSC-derived exosomal miR-4461 inhibits CRC cell proliferation, migration, and invasion by reducing COPB2 expression, suggesting that miR-4461 may be a potential target for diagnosis and treatment of CRC [136]. In addition, MSC-exosome-derived miR-3940-5p inhibits CRC cell invasion, EMT, and tumor growth and metastasis by targeting ITGA6 and subsequent TGF-*β*1 inactivation [137]. ADMSC-derived EVs carrying miR-15a inhibit the immune escape of CRC cells by the downregulation of the KDM4B/HOXC4/PD-L1 axis [138].

In addition to the anticancer effect of MSC exosomal miRNAs, Luetzkendorf and colleagues showed that human MSCs could be induced by the third-generation lentiviral vector system to produce TRAIL- (tumor necrosis factor-related apoptosis-inducing ligand-) MSCs. It has been demonstrated that TRAIL-MSCs can reduce tumor growth of CRC cells in vivo by inducing apoptosis [139]. Another study that supported the hypothesis showed that TRAIL-MSCs can induce the apoptosis of TRAIL-CRC-resistant cells and overcome the tumor resistance to TRAIL in clinical treatment, suggesting that MSCs can be used as a carrier for clinical cancer treatment [140]. Zheng et al. revealed that CXCR4 overexpression by BM-MSCs increases the ability of stem cells to nest in the intestinal tract and improves colitis-related tumorigenesis in mice [141]. Due to the limited data available, the clinical application of MSCs in the treatment of CRC remains controversial. The issue of the clinical therapeutic value of MSCs is an intriguing one that could be usefully explored in further research to promote the development of stem cell therapies.

### 5.2. Promoting Effects on CRC

Tumor-stromal interaction plays a key role in the biology of CRC. With the deepening of research on MSCs, recent studies show that MSCs are recruited from the bone marrow into tumor stroma and form an important component of the tumor microenvironment, being the main source of CAFs. It is involved in the regulation of intestinal inflammation, epithelial proliferation, stem cell maintenance, angiogenesis, and extracellular matrix remodeling and metastasis [142]. Wu et al. illustrated that the protumor effect of MSCs is attributable to the altered expression of cyclin and the inhibition of apoptosis, possibly through the AMPK/mTOR-mediated activation of NF-*κ*B to promote the progression of CRC [143]. Similarly, Zhang and colleagues found that human CRC-derived MSCs promote the progression of CRC cells through IL-6/JAK2/STAT3 signaling and activate PI3K/AKT signaling [144]. A recent study showed that TGF-*β*1 can induce the differentiation of MSCs to CAFs through the activation of the JAK/STAT3 signaling pathway and promote migration and invasion of CRC cell lines HCT116 and HT29 cells [145]. Tan et al. revealed that the CXCR4/TGF-*β*1 axis plays an important role in the transformation of the tumor microenvironment by mediating the differentiation of MSCs to CAFs, promoting the growth and metastasis of CRCs [66].

In addition, studies by Nishikawa and colleagues have shown that MSCs interact with CRC cells through CCL3/4/5-CCR5, thereby promoting the growth of CRC tumors in vivo [146]. Chen et al. found that inflammation-activated human MSCs promote the epithelial-mesenchymal transformation (EMT) process and progression of CRC through the CCL5/*β*-catenin/Slug pathway [147]. Similarly, it is reported that BM-MSCs are implanted in nude mice after subcutaneous injection of HCT116-cancer stem cells (CSCs) to construct xenograft tumors. BM-MSCs can promote the migration and invasion of CSCs in CRC, suggesting that it can be a potential therapeutic target for CRC [148]. Further studies by De Boeck and colleagues found that BM-MSCs stimulate the invasion, survival, and tumorigenesis of CRC cells by releasing soluble NRG1 and activating HER2/HER3-dependent PI3K/Akt signaling cascade in CRC cells, and the high expression of NRG1 is associated with poor prognosis [149].

In a study by Widder and colleagues, MSC-CRC interaction promoted the formation of three-dimensional globules in CRC cells with a dysfunctional E-cadherin system. Further analysis showed that MSCs may affect the early xenotransplantation growth of specific *α*-catenin-deficient CRC cells by secreting extracellular matrix and ultimately play a cancer-promoting role [150]. EMT is an important mechanism for the progression of CRC, and SPARC is an important EMT-related factor in CRC, which is involved in the interaction between tumor cells and stromal cells. Naito and colleagues confirmed that MSCs induce the tumor-stromal formation and EMT process by expressing secreted protein acidic and rich in cysteine (SPARC) [151] and demonstrating a stronger ability to attack peripheral tissues through the mediation of newly expressed surface TGF-*β* on MSCs after coculture with tumor cells [152], thereby promoting the occurrence and development of CRC.

In addition to MSCs playing a role in the proliferation, invasion, and migration of CRC cells, Li and colleagues also found that MSCs can regulate the P53/P21 pathway through posttranscriptional regulation to help CRC resist senescence [153]. MSCs promote CRC angiogenesis and tumor growth through high levels of paracrine proangiogenic factor IL-8 [154]. Interestingly, besides the regulatory effects of MSCs on CRC cells, CRC cells can also induce morphological and functional changes of colon MSCs through secretion of exosomes, which is conducive to the growth and malignant progression of tumors [155], confirming the mutual promotion between the two. In addition to MSCs promoting the EMT process of CRC through direct cell-to-cell contact [156], recent studies by Li and colleagues found that miR-222 carried by extracellular vesicles derived from MSCs targets ATF3 binding and suppresses the transcriptional activity of AKT1, thereby promoting malignant invasion and immune escape of CRC cells [157].

## 6. Conclusion

Concerning CRC, various factors such as diet, environment, and genetic susceptibility greatly influence the constantly increasing incidence of CRC. The current cure rate of CRC is low, and the available treatment interventions are associated with a lot of side effects. MSC therapy has been highly regarded as a promising method for the treatment of clinical diseases including tumors. They have excellent conditions for the treatment of CRC due to their low immunogenicity, strong immune regulatory function, self-renewal ability, and easy accessibility. MSC-derived exosomes also express certain RNAs that participate in the inhibition of CRC growth and progression. These vesicles could also be engineered to serve as effective carriers of drugs and other therapeutic molecules to the CRC cells. However, like other malignancy studies, research on the application of MSCs in CRC is confronted with many challenges including the complex pathogenesis of CRC, the dual regulatory effects of MSCs on CRC, and the uncertainties of therapeutic dosage, administration mode, and adverse reactions shown in certain studies. Therefore, it is imperative to explore solutions to these hindrances, in addition to further investigating the regulatory mechanism and cargo sorting, functional modification, and carrier potentials of MSCs in CRC therapy for novel treatment interventions.

## Figures and Tables

**Table 1:** The sources and characteristics of MSCs.

Name	Source	Surface marker	Separation	Reference
BM-MSCs	Bone marrow	Stro1(+), Stro4(+), CD271(+), CD146(+), CD106(+), CD73(+), CD105(+), SSEA3(+), FZD9(+), SUSD2(+), LEPR(+), GD2(+), 3G5(+), CD90(+), CD140b(+), CD340(+), CD349(+), CD44(-), CD31(-), CD34(-), CD45(-), Lin(-), CD140a(low/-)	Prospective isolation based on monoclonal antibodies Density-gradient centrifugation	[36–40]
AD-MSCs	Adipose tissue	CD271(+), CD146(+), TM4SF1(+), CD44(+), CD73(+), CD90(+), CD105(+), CD36(+), CD163(+), CD200(+), CD273(+), CD274(+), TM4SF1(+), CD24(+), CD140B(+), CD34(-), Stro-1(-), SSEA-4(-)	Enzymatic digestion Mechanical centrifugation	[39–43]
UCB-MSCs	Umbilical cord blood	CD105(+), CD73(+), CD90(+), GD2(+), SSEA-4(+/-), CD146(+), CD49f(+), PODXL(+), TM4SF1(+), Stro1(-), CD45(-), CD34(-), CD14(-), HLA-DR(-), CD79a(-), CD11b(-), CD271(-), CD19a(-)	Density-gradient purification	[39, 40, 44]
UC-MSCs	Umbilical cord	CD146(+), CD29(+), CD44(+), CD73(+), CD90(+), CD105(+), Stro-1(-), CD271(-), SSEA-4(-)	Enzyme digestion	[39, 45, 46]
eMSCs	Endometrium	(PDGFR*β*)/CD140b(+), CD146(+), SUSD2(+), CD29(+), CD44(+), CD73(+), CD90(+), NTPDase2(+), CD105(+), CD31(-), CD34(-), CD45(-), Stro-1(-)	Enzyme digestion	[39, 47–50]
MenSCs	Menstrual blood	CD56(+), CD73(+), CD90(+), CD105(+), CD146(+), SSEA-4(+)	Isolated cells were seeded into culture flasks	[51]
dPSCs	Dental pulp	CD9(+), CD10(+), CD13(+), CD29(+), CD44(+), CD59(+), CD73(+), CD9(+), CD105(+), 3G5(+), CD106(+), CD146(+), Stro-1(+), SSEA-4(+), CD166(+), CD271(+), CD14(-), CD19(-), CD24(-), CD31(-), CD34(-), CD45(-), CD117(-), CD133(-)	Enzymatic digestion of the pulp tissue Explant method	[39, 52, 53]

**Table 2:** The general clinical application of MSCs.

Systems	Disease	Effect	Mechanism	Reference
Respiratory system	Bronchopulmonary dysplasia; non-small cell lung cancer; ARDS; asthma inflammation; diabetic lung fibrosis	Improve lung function; reduce pulmonary fibrosis; relieve pulmonary hypertension	Increase the “M2” macrophages; mitochondrial transfer; adjusting Sirt3-mediated responses; exosomal transfer of miR-144	[70, 86–89]
Digestive system	IBD; intestinal ischemia-reperfusion injury (IRI)	Suppression of inflammatory responses; improve I/R-induced intestinal damage; improve gut barrier function	IL-10; macrophage polarization; TSG-6 through hyaluronan-CD44 interactions in an Akt-dependent manner; promote Claudin-3, Claudin-2, and ZO-1 expression; NLRP3-related signaling pathways	[90–94]
Endocrine system	Type 2 diabetes mellitus (T2DM)	Reduce blood glucose levels; reverse insulin resistance	Inhibition of STZ-induced *β*-cell apoptosis; activation of autophagy via the AMPK pathway; blockade of the NLRP3 inflammasome activation	[95–97]
Immune system	Rheumatoid arthritis; systemic lupus erythematosus (SLE); allergic asthma	Reduce joint destruction; improve the immune system	Restore the balance between memory T cells populations; miRNA-150-5p; release TGF-*β*1 to generate CD4 + CD25 + Foxp3 + T-reg cells; expand IL-10 producing lung interstitial macrophages	[98–102]
Nervous system	Stroke; neuroinflammation	Improve neurological impairment and long-term neuroprotection; attenuate neuroinflammation	Inhibiting STAT3-dependent autophagy; microRNA cluster miR-17-92	[103–107]

**Table 3:** The different treatment options in CRC.

Therapeutic method	Effects	Reference
Surgery	The cornerstone of CRC treatment	[120]
Chemotherapy	Prolongs survival and improves symptoms and quality of life	[121]
Radiotherapy	Achieves local control and improves long-term prognosis	[122]
Targeted therapy	Reduces potential liver metastasis associated with CRC (antiviral therapy)	[123]
Immunotherapy	Achieves long-term durable remission in patients	[124, 125]
Probiotics	Enhance the immune barrier, regulate the intestinal immune state, inhibit pathogenic enzyme activity, regulate CRC cell proliferation and apoptosis, regulate redox homeostasis, and reprogram intestinal microbial composition	[126]
Prebiotics	Stimulate the growth and/or activity of specific bacteria in the gut, improve host health, possess prebiotic potential, modulate gut microbiota composition, a product of fermentation metabolites, antiadhesive properties against pathogens, and alter the gene expression profile	[127]
Postbiotics	Modulate the composition of the gut microbiota and the functionality of the immune system, promote the CRC treatment effectiveness, and reduces its side effects in CRC patients	[128]
Antibiotics	Improve the treatment efficacy of oxaliplatin-based therapy and reduce cancer severity through controlling *F. nucleatum*	[129, 130]
Nonsteroidal anti-inflammatory drugs (NSAIDs)	Prolong survival time	[131]

Fecal microbiota transplantation (FMT)	Restores the sensitivity of patients to anticancer drugs and enhances the immune response	[132]

**Table 4:** The effects of MSCs within the CRC microenvironment.

MSC-CRC interaction	Mode of function	Mechanisms	References
Inhibition	Cell-to-cell contact	Inhibiting the expression of proinflammatory cytokines and STAT3 activation	[133]
	Cell-to-cell contact	Induces apoptosis and interferes with tumor initiation through the dysregulation of Wnt and TGF-*β*-Smad signaling pathways	[134]
	Paracrine	miR-165-p overexpression in BM-MSC-exosomes inhibited the proliferation, migration, and invasion and promoted apoptosis of CRC cells by downregulating ITGA2 expression.	[135]
	Paracrine	miR-4461 in BM-MSC-exosomes inhibits the proliferation, migration, and invasion of CRC cells by reducing the expression of COPB2.	[136]
	Paracrine	MSC-exosome-derived miR-3940-5p inhibited CRC cell invasion, EMT, and metastasis by targeting ITGA6 and subsequent TGF-*β*1 inactivation.	[137]
	Paracrine	miR-15a carried by AD-MSC-EVs restricted CRC immune escape by downregulating the KDM4b/HOXC4/PD-L1 axis.	[138]

Promotion	Cell-to-cell contact	Regulating cell cycle and inhibiting apoptosis through the activation of NF-*κ*B mediated by AMPK/mTOR	[143]
	Cell-to-cell contact	Promoting the progression of CRC cells through IL-6/JAK 2/STAT3 signal	[144]
	Cell-to-cell contact	The activation of JAK/STAT3 stimulated by the TGF-*β*1 or CXCR4/TGF-*β*1 axis can induce MSCs to differentiate into CAFs, which can promote the progression of CRC.	[66, 145, 152]
	Cell-to-cell contact	Interacting with CRC cells through CCL3/4/5 -CCR5 to promote the growth of CRC tumors in vivo	[148]
	Cell-to-cell contact	Promoting the EMT process of CRC through the CCL5/*β*-catenin/Slug pathway and SPARC	[147, 151]
	Cell-to-cell contact	Activating the HER2/HER3-dependent PI3K/Akt signaling cascade in CRC cells by releasing soluble NRG1.	[149]
	Cell-to-cell contact	Affecting the early xenograft growth of CRC cells with specific *α*-catenin deficiency by secreting extracellular matrix	[150]
	Cell-to-cell contact	Regulating the P53/P21 pathway through posttranscriptional regulation helps CRC resist senescence.	[153]
	Cell-to-cell contact	Promoting CRC angiogenesis through paracrine's high levels of the proangiogenic factor IL-8	[154]
	Paracrine	miR-222 targets ATF3 and inhibits the transcriptional activity of AKT1, thereby promoting malignant invasion and immune escape of CRC cells.	[157]

Affection	Paracrine	Inducing morphological and functional changes in colon mesenchymal stem cells by secreting exosomes	[155]
